# Study of the acoustic stem evoked potentials in blood circulation disorder in the vertebral basilar basin

**DOI:** 10.1186/cc10903

**Published:** 2012-03-20

**Authors:** I Vlasova, T Vizilo, V Tsiuriupa

**Affiliations:** 1 Scientific Clinical Center of Miners' Health Protection, Leninsk-Kuznetsky, Russia

## Introduction

Acoustic brainstem evoked potentials (ABEP) offer a possibility to objectivise disorder of the brain stem structure function.

## Methods

There were flicks of 9.5 Hz with intensity 70 dB higher than the hearing threshold. The latency time of the I to V peaks, the interpeak intervals (IPI), the peak amplitudes (PA) and the amplitude correlations were measured. The clinical neurophysiological assessment of 30 patients (16 men and 14 women, age from 40 to 70 years) with clinical presentation of ischemic stroke in the vertebral basilar basin (VBB) allowed us to determine the following forms of acute ischemic disorders of the brain circulation: transitory ischemic attacks (TIA) (*n *= 16), lacunar infarction (LI) (*n *= 10), and nonlacunar infarction (NLI) in VBB (*n *= 4).

## Results

According to the ABEP the common feature in all groups of patients was the decrease of the correlation of the V PA to I PA that was significant in 56% cases in NLI, in 47% cases in LI and in 15% cases in TIA; the decrease of all PA (to 0.12 to 0.15 mkV) was significant in 49% cases in NLI and in 39% cases in LI. A distinct tendency to the laterality of the peak latency increase in TIA and LI in 49% of cases, and a significant laterality of the peak latency increase in 35% that reflected the dissymmetric disorder of the neuronal acoustic activity of the brainstem were observed. There was a tendency to increase of the I to III and III to V intervals in 46 to 61% in TIA. The I to III and III to V IPI were significantly increased in LI and NLI, in 35% and 47% cases respectively. The patients with NLI demonstrated an increase of the I to V IPI. There was such neurophysiological dynamics. The reconstruction of the amplitude and peak latency in TIA was observed in 100% of cases in the treatment process. This was not registered in LI and NLI.

## Conclusion

All strokes in the VBB are characterized by functional changes on the part of the brain stem structures predominantly at the pontomedullary and pontomesencephalic levels. There is a dependence between stroke severity, brainstem structure damage and neurophysiological dynamics. ABEP allow one to objectivise the brain stem structure dysfunction in the VBB's disturbed circulation.

